# Tauroursodeoxycholic Acid Protects Nucleus Pulposus Cells from Compression-Induced Apoptosis and Necroptosis via Inhibiting Endoplasmic Reticulum Stress

**DOI:** 10.1155/2018/6719460

**Published:** 2018-03-12

**Authors:** Wenzheng Wang, Xiangcheng Qing, Baichuan Wang, Kaige Ma, Yulong Wei, Zengwu Shao

**Affiliations:** Department of Orthopaedics, Union Hospital, Tongji Medical College, Huazhong University of Science and Technology, Wuhan 430022, China

## Abstract

Tauroursodeoxycholic acid (TUDCA) is a kind of hydrophilic bile acid, which could protect cells from death via inhibiting endoplasmic reticulum (ER) stress. However, the role of TUDCA in compression-induced intervertebral disc degeneration (IVDD) has not been elucidated. Here, we used a previously described device to mimic* in vivo* compression conditions. NP cells treated with DMSO or TUDCA were exposed to compression. Then, cell viability, morphology, and apoptosis were detected. Furthermore, apoptosis-related proteins and necroptosis markers were detected too. To investigate the specific cytoprotective mechanisms of TUDCA in IVDD, we detected the ER morphology by electron microscopy. In addition, the ER stress of nucleus pulposus (NP) cells was quantitatively evaluated by analyzing the level of ER-stress-associated proteins. Our results revealed that TUDCA could protect NP cells from excessive compression-induced death by reducing the apoptosis and necroptosis. In addition, ER stress is involved in pathogenesis of IVDD induced by excessive compression and plays a detrimental role. TUDCA exerts its protective functions by inhibiting ER stress. In conclusion, TUDCA could protect NP cells from compression-induced death, which suggested that treatment by TUDCA may be a potential method to retard IVDD.

## 1. Introduction

Intervertebral disc degeneration (IVDD) is responsible for approximately 40% of low back pain in human [1], which immensely causes society burden in the world. Hence, the research related to the etiology and cure of IVDD is essential. The intervertebral disc (IVD) is a load-bearing component of human body, which is subjected to various mechanical load types. Previous studies have displayed that appropriate mechanical stimulus can exert a protective effect on the IVD [2, 3], while excessive mechanical force is proven to be detrimental for IVD [4, 5]. Excessive compression leads to disc degeneration by inducing cell death and extracellular matrix (ECM) loss [6, 7], both of which are central pathological mechanisms for IVDD. However, the exact molecular mechanism by which excessive compression modulates degenerative progression is still not clear.

Tauroursodeoxycholic acid (TUDCA) is a kind of hydrophilic bile acid that is normally produced endogenously in humans at extremely low levels [8]. Multiple studies have demonstrated that TUDCA could protect cells from ER-stress-induced death in numerous human diseases such as type 2 diabetes, osteoarthritis, and acute pancreatitis [8, 9]. In addition, accumulating evidence indicates that TUDCA exerts its biological functions mainly by acting as endoplasmic reticulum (ER) chaperone that could inhibit ER stress and block the activation of unfolded protein response (UPR) [10]. However, the effects of TUDCA on IVDD have not been elucidated before.

The ER is the major organelle responsible for the proper folding of secreted and organelle-targeted proteins. ER stress is referred to as the condition in which cells appear to perform near the functional limits of their secretory pathway capacity and the load imposed on the ER protein-folding machinery overwhelms its capability [11]. Several physiological and pathological stimuli cause the accumulation of unfolded proteins in the ER lumen, disrupt ER homeostasis, and trigger ER stress [11, 12]. The unfolded protein response (UPR) is a conserved signal transduction pathway that is activated when cells fail to meet the protein-folding demands under ER stress. It is mediated by three ER-localized transmembrane proteins: double-stranded-RNA-dependent protein kinase- (PKR-) like ER kinase (PERK), activating transcription factor 6 (ATF6), and inositol-requiring enzyme 1 (IRE1). All three UPR transducers are conjugated with the ER-resident chaperone GRP78 and maintained in a nonactive form in resting cells. When ER stress is triggered, GRP78 disaggregates and the UPR pathways are activated [13]. The accumulation of unfolded proteins may be decreased via the attenuation of global mRNA translation and the upregulation of ER chaperones. However, when cells are exposed to prolonged ER stress, they are not able to remove incorrectly folded proteins from the ER and programed cell death is induced. The UPR pathways activate downstream molecules, such as JNK (c-Jun N-terminal kinase) and CHOP (C/EBP homologous protein) and thus induce caspase-4/-12-mediated apoptosis [14]. Accordingly, ER stress can play either a protective or a detrimental role in various diseases. ER stress is involved in multiple diseases. In cancer, ER stress can protect tumor cells from apoptosis, promote angiogenesis, and increase the drug resistance of tumors [15]. The core pathogenesis of neurodegenerative disease is the inefficiency of cells to activate UPR and ER-associated degradation [16]. For IVDD, ER-stress-induced disc cell apoptosis was found to be important for the pathogenesis of IVDD [17]. Furthermore, ER stress inhibitors such as TUDCA have been successfully applied for the treatment of some metabolic diseases [18, 19]. For IVDD, however, whether TUDCA could inhibit compression-induced ER stress in NP cells is still unknown.

Necroptosis is a kind of programmed cell death which shares some common morphological features as necrosis, including the disruption of cell membrane, swelling of organelles, and condensation of chromatin [18]. Necroptosis is a caspase-independent process that is triggered by death receptors, which requires the kinase activity of receptor-interacting protein kinase 1 (RIPK1) and receptor-interacting protein kinase 3 (RIPK3). Necroptosis participates in the pathogenesis of many diseases, including ischemic injury, neurodegeneration, and acute chest syndrome [20]. Our recent study also revealed that necroptosis is involved in the compression-induced cell death of IVDD [21]. Therefore, inhibiting the necroptosis of NP cells may be a potential target for the treatment of IVDD.

In the current study, we first focused on the effects of TUDCA on the survival, cell morphology, apoptosis, and necroptosis of rat NP cells under compression. Furthermore, to determine whether TUDCA exerts its biological functions via inhibiting the ER stress, we observed ER morphology and detected the ER stress level of NP cells for various durations of a compressive force stimulus. Our study revealed protective effects of TUDCA on NP cells for the first time.

## 2. Methods

### 2.1. NP Cells Isolation and Culture

The experimental protocol was approved by the animal experimentation committee of Huazhong University of Science and Technology. Sprague-Dawley rats (10 weeks old, male) were purchased from the Experimental Animal Center of Tongji Medical College, Huazhong University of Science and Technology. Briefly speaking, rats were euthanized by anesthesia, and the lumbar spines were excised under aseptic conditions. The vertebral appendices and the surrounding soft tissues were thoroughly removed. After the discs were transected from the front edge, the NP tissue was isolated using ophthalmic forceps. The harvested NP tissue was cut into 1 mm^3^ fragments, and then the fragments were digested with 0.25% collagenase type II (Invitrogen, Carlsbad, CA, USA) at 37°C for 10 min and filtered through a 70 *μ*m filter to remove debris. The obtained cells were seeded in a 6-well culture plate and incubated in Dulbecco's modified Eagle's medium/Ham's F-12 (Gibco, Waltham, MA, USA) with 20% fetal bovine serum (Gibco) at 37°C and 5% CO_2_. Cells incubated with 10% fetal bovine serum at the second passage and maintained in a monolayer were used throughout the experiments.

### 2.2. Application of a Compression Apparatus to Rat NP Cells

**Figure 1:**
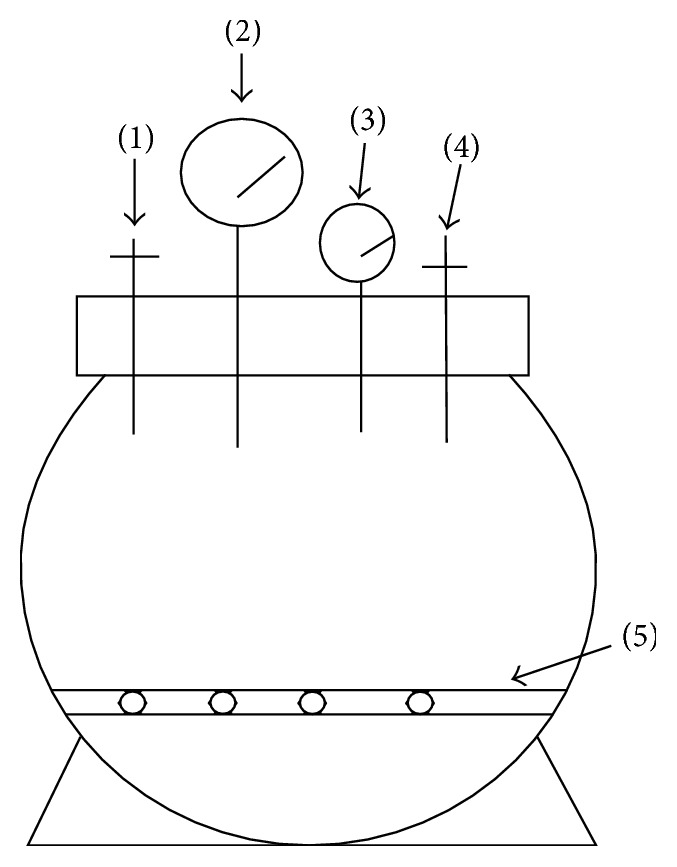
*Schematic illustration of the custom-made compression apparatus*. (1) Air inlet/outlet. (2) Thermometer. (3) Barometer. (4) Safety valve. (5) Bracket.

To mimic* in vivo* conditions, cells were cultured in a previously described compression apparatus made of stainless steel [22]. The pressure apparatus (capacity, 8.2 L) consisted of a sealed can and two meters was constructed to withstand up to 1 MPa of pressure (Figure 1). Rat NP cells were placed in cell culture plates or dishes as monolayer and positioned on the bracket in the sealed can. Sealed can was filled with a small quantity of distilled water at the bottom to maintain moisture. After the sealed can has been fastened down, the compressed gas was pumped into the container until the barometer reached 1 MPa. The gas was mixed with 0.5% CO2 + 9.5% air + 90% N2 to make sure the partial pressure of CO2 and air was the same as it was in normal cell culture incubators. At last, the compression apparatus was housed in a cell culture incubator during experiments to maintain a constant temperature of 37°C.

### 2.3. Transmission Electron Microscopy

After the indicated treatments, cells in different groups were harvested and fixed in phosphate-buffered saline (PBS) (pH = 7.4) containing 2.5% glutaraldehyde for 2 h at room temperature and then postfixed by use of 1% osmic acid for 1 h at room temperature. The samples were dehydrated in graded ethanol and embedded in epoxy resin. Then, the samples were cut into 70 nm sections using the Ultracut UCT Ultramicrotome (Leica, Wetzlar, Germany). Each section was stained with uranyl acetate and lead citrate for 10 min and observed using the Tecnai G2 12 Transmission Electron Microscope (FEI, Eindhoven, Netherlands).

### 2.4. Cell Counting Kit-8 (CCK-8) Assays

NP cells were cultured in 96-well cell culture plates. The seeding density was 5 × 10^3^ cells per well. After 24 h, the original culture medium was changed to the culture medium plus 0.5 *μ*l of TUDCA at different concentrations or 0.5 *μ*l of DMSO for the control group. The cells were then cultured in the compression apparatus for 18 h. Following the manufacturer's instructions, 10 *μ*l of CCK-8 solution (Dojindo, Kumamoto, Japan) and 100 *μ*l of serum free medium were added to each well and incubated for 2 h at 37°C. The absorbance of each well was determined using the Infinite F50 Microplate Reader (TECAN, Männedorf, Switzerland) at 450 nm.

### 2.5. Hoechst 33258 Staining for the Observation of Apoptotic Cell Nuclei

Cells were seeded in 6-well cell culture plates at a density of 10^5^ cells per well. After treatment with TUDCA at different concentration or DMSO for the control group, cells were immediately cultured in the compression apparatus for 18 h. Then, cells of each group were fixed with 4% paraformaldehyde for 10 min and washed with PBS. The cells were stained with 1 *μ*g/ml Hoechst 33258 (Beyotime, Haimen, China) in PBS for 5 min in the dark. Morphological changes in the nuclei of apoptotic cells were evaluated and photographed under the IX81 Fluorescence Microscope (Olympus, Tokyo, Japan). The excitation wavelength was 350 nm and the emission wavelength was 460 nm.

### 2.6. Measurement of Apoptosis by Flow Cytometry and Fluorescence Microscopy

The apoptosis of NP cells was detected using the Annexin V-FITC/PI Apoptosis Detection Kit (Beyotime) and fluorescence microscope. For flow cytometry analysis, NP cells were cultured in 6-well cell culture plates. After treatment with TUDCA or DMSO and culturing in the compression apparatus for 18 h, the total cells including those attached to the plate and those suspended in the culture medium of each group were harvested by trypsinization and centrifugation. Then, cells were washed with PBS and resuspended in a commixture, including 200 *μ*L of binding buffer, 5 *μ*L of FITC-conjugated Annexin V, and 10 *μ*L of propidium iodide (PI) and incubated for 10 min at room temperature in dark conditions. Apoptosis was detected by the FACalibration flow cytometer (Becton Dickinson, Franklin Lakes, NJ, USA). For fluorescence microscopy observation, compression-treated cells were stained with Annexin V-FITC/PI according to manufacturer's instructions. The double-positive cells after staining with Annexin V-FITC and PI were regarded as apoptotic cells and cells negative for both were nonapoptotic. Apoptotic cells were quantified and represented as the percentage of the total cell count.

### 2.7. Western Blot Analysis

Cells of various groups were collected and washed with ice-cold PBS. RIPA lysis buffer (50 mM Tris, 150 mM NaCl, 1% NP-40, 0.5% sodium deoxycholate, and 0.1% SDS, Beyotime) plus phenylmethanesulfonyl fluoride (Beyotime) and phosphatase inhibitor cocktail I (Sigma, St. Louis, MO, USA) were used for total protein extraction. The Enhanced BCA Protein Assay Kit (Beyotime) was used to detect the protein concentration of the lysates. Equivalent amounts of protein were loaded onto each well, separated by 10% or 15% SDS-PAGE, and then transferred to polyvinylidene fluoride membranes (Bio-Rad, Hercules, CA, USA). The membranes were blocked with 5% bovine serum albumin (Beyotime) in TBST (a mixture of Tris-buffered saline and Tween-20) for 1 h at room temperature and incubated overnight at 4°C with primary antibodies against p-PERK (#3179; Cell Signaling Technology (CST), Beverly, MA, USA), p-IRE1 (#ab48187; Abcam, Cambridge, UK), p-eIF2*α* (#3398S; CST), CHOP (#2895p; CST), Cleaved caspase-12 (#ab62484; Abcam), RIPK1 (SAB3500420; Sigma-Aldrich, Germany), p-RIPK1 (#9621; CST), RIPK3 (#ab62344; Abcam), p-RIPK3 (#ab195117; Abcam), and GAPDH (Boster, Wuhan, China). After incubation with secondary antibodies (Boster) labeled with horseradish peroxidase for 1 h at room temperature, immunoreactive bands were developed using BeyoECL Plus (Beyotime). The intensity of bands was quantified using Gel-Pro application.

### 2.8. Statistical Analysis

Statistical analysis was performed in IBM SPSS Statistics 19. All data obtained from at least three independent assays were presented as means ± SD. Student's* t*-test was used to analyze differences between the means of two groups. For the CCK-8 assay and the flow cytometry analysis after Annexin V/PI double staining, data was analyzed by one-way ANOVA followed by least significant difference (LSD) tests to analyze differences between control and treatment groups. *P* < 0.05 was considered significant.

## 3. Results

### 3.1. TUDCA Protects NP Cells from Compression-Induced Death

**Figure 2:**
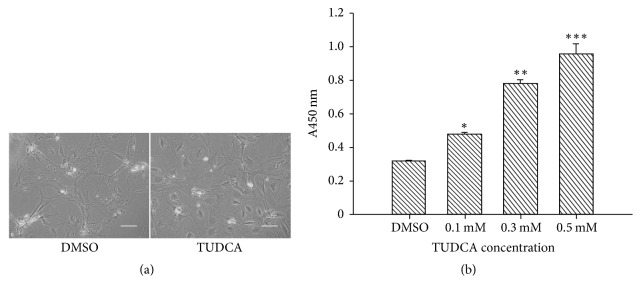
Effects of TUDCA on compression-induced NP cells death. (a) Phase-contrast photomicrographs of morphological differences between the DMSO group and high-dose TUDCA group (magnification ×400, scale bars represent 50 *μ*m). (b) The number of survival NP cells under compression stimulation was detected by CCK-8 assays. Data are expressed as means ± SD of three independent experiments (^*∗*^*P* < 0.05, ^*∗∗*^*P* < 0.01, and ^*∗∗∗*^*P* < 0.001 versus DMSO control).

To determine whether TUDCA has protective effects on compression-induced death of NP cells, cell morphology observation and CCK-8 assay were applied. Different concentrations of TUDCA (0.1, 0.3, and 0.5 mM) were used for the low-, mid-, and high-dose groups. Cells treated with isovolumetric DMSO were set as the control group. In each experiment group, TUDCA was dissolved in isometric DMSO as control group to exclude the side effect of DMSO. As expected, after exposure to compression (1 MPa for 18 h), there were obvious morphological differences between the group treated with 0.5 mM TUDCA and the group treated with DMSO. The cells in the DMSO group were shriveled and contact area between cells and the culture plate was reduced; some cells even began to shed from the plate, whereas cells in TUDCA group showed healthier cell morphology (Figure 2(a)). In addition, the CCK-8 results further confirmed that TUDCA treatment could dramatically inhibit compression-induced cell death of NP cells, and this effect was concentration-dependent (Figure 2(b)). Based on above results, we could conclude that TUDCA had protective effects on NP cells under compression.

### 3.2. TUDCA Decreases the Apoptosis of NP Cells Induced by Compression

**Figure 3:**
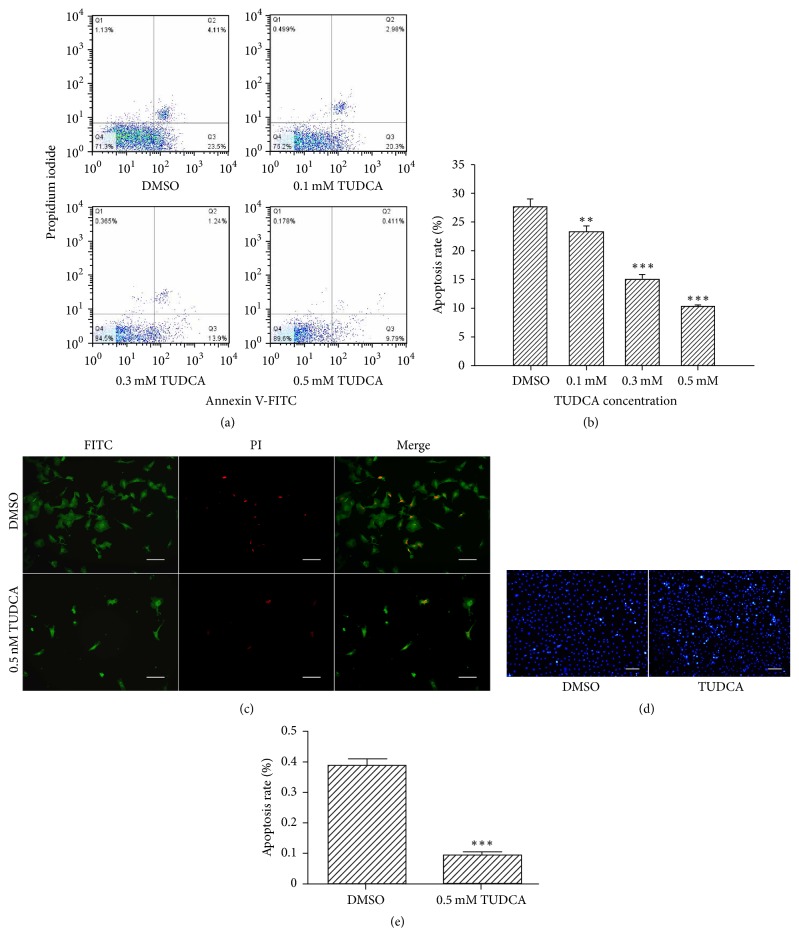
The influence of TUDCA on compression-induced apoptosis of NP cells. (a) Dot graphs obtained by flow cytometry analysis after Annexin V-FITC/PI dual staining. Early apoptotic cells were Annexin V-FITC (+) and PI (−), late apoptotic cells and necrotic cells were Annexin V-FITC (+) and PI (+), and undamaged cells were Annexin V-FITC (−) and PI (−). (b) The apoptosis rate for each group. Data are expressed as means ± SD of three independent experiments (^*∗∗*^*P* < 0.01 and ^*∗∗∗*^*P* < 0.001 versus DMSO control). (c) Fluorescence photomicrograph of apoptotic cells after Annexin V-FITC/PI dual staining (magnification ×200, scale bars represent 100 *μ*m). The fluorescence photomicrographs of cells stained with FITC (green) and PI (red) were taken under the same visual field and then merged using Image-Pro Plus software. (d) Fluorescence photomicrograph of apoptotic nuclei after Hoechst 33258 staining. Apoptotic nuclei were brightly stained by Hoechst 33258 (magnification ×100, scale bars represent 200 *μ*m). (e) Statistical analysis of the apoptotic rate after Hoechst 33258 staining (^*∗∗∗*^*P* < 0.001 versus DMSO control).

Apoptosis of NP cells plays important roles in the pathogenesis of IVDD. To explore whether TUDCA could inhibit apoptosis induced by compression, cells were treated as described above and the rate of apoptosis was measured by flow cytometry analysis. Our results demonstrated that, compared with control group, the proportions of early apoptosis cells (Q3) and late apoptosis cells (Q2) as well as the total apoptosis rate were all decreased by TUDCA treatment in a dose-dependent manner (Figures 3(a) and 3(b)). Fluorescence photomicrographs of Annexin V-FITC/PI dual-stained cells also clearly revealed a decrease in apoptotic cells (Figure 3(c)). Moreover, as shown in the photomicrograph of Hoechst 33258-stained cells, in contrast to the control group, the proportion of brightly stained nuclei was diminished by TUDCA (Figure 3(d)). The apoptotic cells in TUDCA group differed significantly compared with the DMSO group (Figure 3(e)).

**Figure 4:**
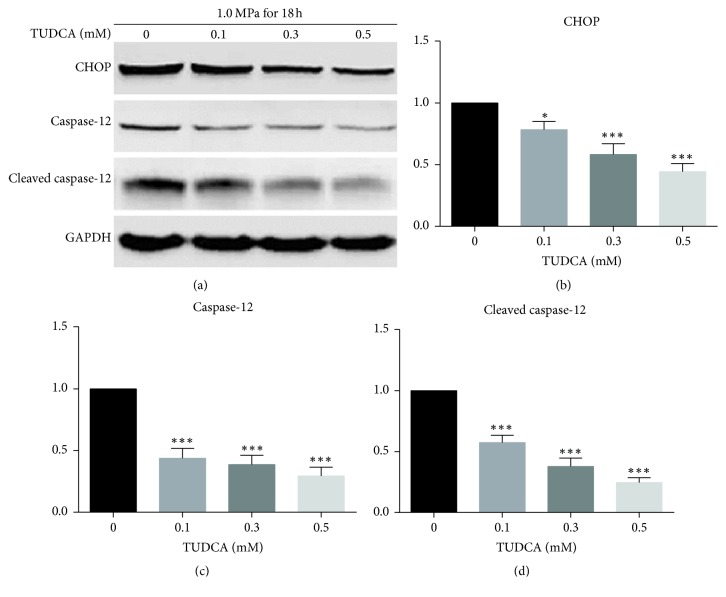
The level of ER-mediated apoptosis-related biomarkers in NP cells treated with DMSO or TUDCA. (a) The Western blot results of ER-mediated apoptosis-related biomarkers CHOP, caspase-12, and cleaved caspase-12. (b), (c), and (d) show the quantitative analysis of expression level of ER-mediated apoptosis-related biomarkers CHOP, caspase-12, and cleaved caspase-12. Results are presented as the fold change in TUDCA-treated groups relative to that in DMSO group (^*∗*^*P* < 0.05 and ^*∗∗∗*^*P* < 0.001 versus DMSO control).

TUDCA mainly acted as an ER chaperone; meanwhile, the ER pathway is one of the three signaling pathways leading to cell apoptosis. So we detected the level of ER-pathway-related proteins CHOP, caspase-12, and cleaved caspase-12 by using Western blot analysis. As shown in the results, the ER pathway of apoptosis was inhibited by TUDCA in a dose-dependent manner (Figure 4). This might partly explain the reason why the cell death was decreased in TUDCA group.

### 3.3. TUDCA Inhibits the Necroptosis of NP Cells Induced by Compression

**Figure 5:**
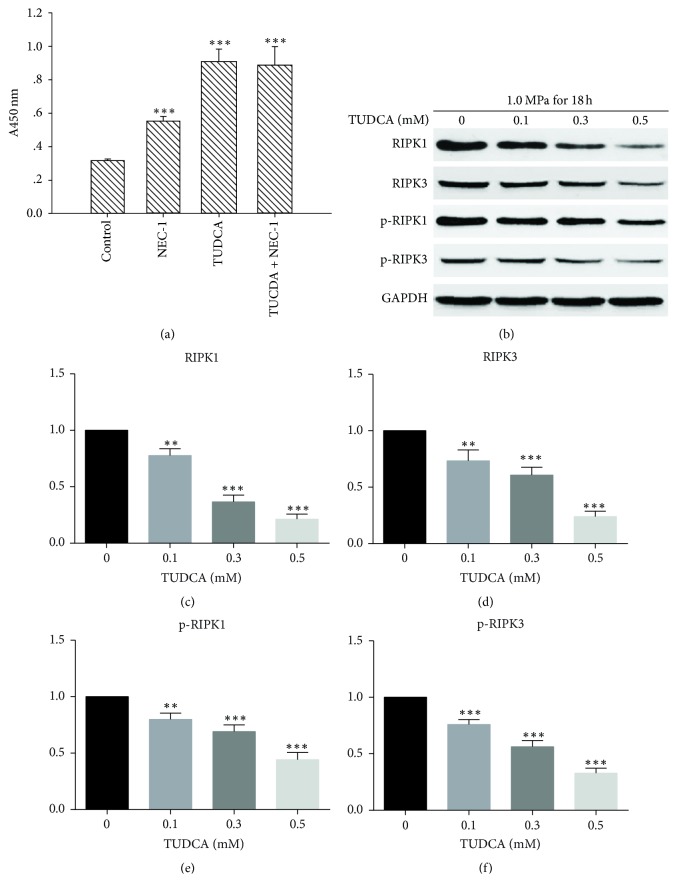
TUDCA reduces compression-induced necroptosis of NP cells in a dose-dependent manner. (a) The NP cells were divided into four groups and the number of survival NP cells was detected by CCK-8 assays. Data are expressed as means ± SD of three independent experiments (^*∗∗∗*^*P* < 0.001 versus control). (b) The Western blot results of necroptosis-related biomarkers RIPK1, RIPK3, p-RIPK1, and p-RIPK3. (c), (d), and (e) show the quantitative analysis of expression level of necroptosis-related biomarkers RIPK1, RIPK3, p-RIPK1, and p-RIPK3. Results are presented as the fold change in TUDCA-treated groups relative to that in DMSO group. (^*∗∗*^*P* < 0.01 and ^*∗∗∗*^*P* < 0.001 versus DMSO control).

Necrostatin-1 (NEC-1) was used as a necroptosis inhibitor because it could particularly inhibit the kinase activity of RIPK1 [18]. To probe the influence of TUDCA on necroptosis, NP cells were divided into four groups: the control group treated with compression only, the group treated with compression and 20 uM NEC-1, the group treated with compression and 0.5 mM TUDCA, and the group treated with compression, NEC-1, and TUDCA. Then, cell viability was detected by CCK-8 assays. As predicted, NEC-1 and TUDCA could, respectively, promote the survival of NP cells. Nevertheless, there was no statistic difference between the cell viability of TUDCA group and TUDCA + NEC-1 group (Figure 5(a)). That means NEC-1 could no longer promote cell viability when TUDCA presented. Therefore, it was logical to speculate that TUDCA could also inhibit the necroptosis of NP cells. To verify our speculation, we detected the level of necroptosis-related biomarkers RIPK1, p-RIPK1, RIPK3, and p-RIPK3 when TUDCA gradient presented. As shown in the results, the necroptosis pathway was inhibited by TUDCA administration in a dose-dependent manner (Figure 5(b)), which explained another reason for the cell viability promoting by TUDCA.

### 3.4. TUDCA Exerts Its Cytoprotective Effects through Inhibiting the ER Stress

**Figure 6:**
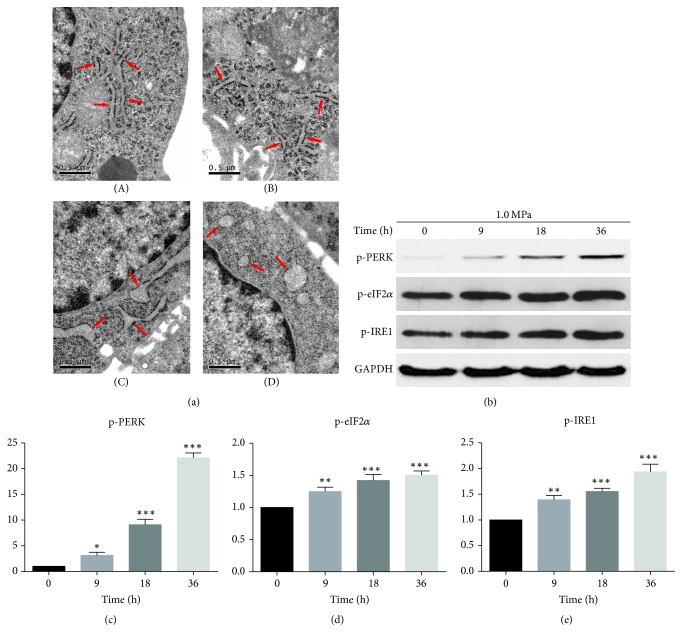
ER stress was triggered in compression-induced NP cell death. (a) Electron microscope photographs of NP cells stimulated by compression for different durations. Control group (A) without exposure to compression. Experimental groups stimulated for (B) 9 h, (C) 18 h, and (D) 36 h. Morphological changes in the endoplasmic reticulum (arrowheads) could be observed by electron microscopy at 26500x. (b) The Western blot results of ER-stress-related biomarkers p-PERK, p-eIF2*α*, and p-IRE1. (c), (d), and (e) show the quantitative analysis of expression level of ER-stress-related biomarkers p-PERK, p-eIF2*α*, and p-IRE1. Results are presented as the fold change in 9, 18, and 36 h groups relative to that in 0 h group (^*∗*^*P* < 0.05, ^*∗∗*^*P* < 0.01, and ^*∗∗∗*^*P* < 0.001 versus 0 h control).

**Figure 7:**
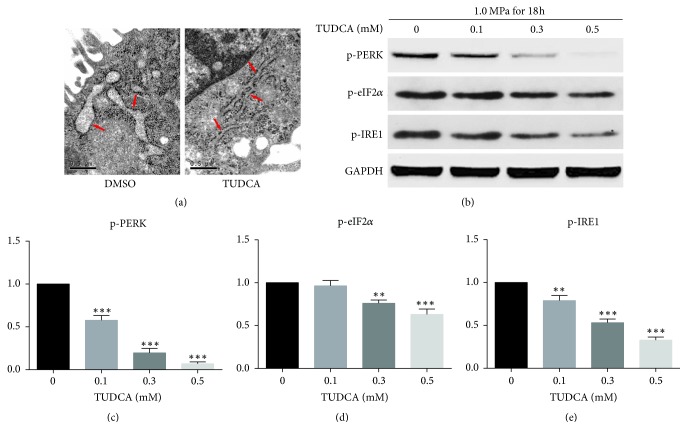
TUDCA inhibits compression-induced ER stress. (a) The ER morphology of the control group and 0.5 mM TUDCA group observed by electron microscope photographs. (b) The Western blot results of ER-stress-related biomarkers p-PERK, p-eIF2*α*, and p-IRE1. (c), (d), and (e) show the quantitative analysis of expression level of ER-stress-related biomarkers p-PERK, p-eIF2*α*, and p-IRE1. Results are presented as the fold change in TUDCA-treated groups relative to that in DMSO group (^*∗∗*^*P* < 0.01 and ^*∗∗∗*^*P* < 0.001 versus DMSO control).

Previous studies have revealed that TUDCA protects cells mainly by inhibiting the ER stress. To clarify whether TUDCA exerts its cytoprotective effects through this mechanism in NP cells too, we first used electron microscopy to examine ER morphological changes. For the morphological changes of ER in compression, as shown in the electron microscope photographs, there were no distinct differences in ER morphology between the 9 h group and the control group. The ER in the 18 h group was swollen but still maintained a basic reticular form. In the 36 h group, the ER exhibited extensive swelling and lost its original reticular form (Figure 6(a)). We then compared ER morphological changes between the control group and 0.5 mM TUDCA group. The ER of the control group was swollen, while the ER morphology of 0.5 mM TUDCA group was almost normal (Figure 7(a)). To further verify whether the ER stress level was influenced by compression and inhibited by TUDCA, we used Western blot analysis to detect the expression of ER stress markers. The results showed that expression levels of p-PERK, p-IRE1, and p-eIF2*α* increased under compression and were positively related to the compression duration (Figure 6(b)). When cells were treated with different concentration of TUDCA, the expression levels of p-PERK, p-IRE1, and p-eIF2*α* were all decreased in a concentration-dependent manner (Figure 7(b)). These results demonstrated that ER stress was triggered in compression-induced IVDD and TUDCA could inhibit the compression-induced ER stress of NP cells in a dose-dependent manner.

## 4. Discussion

Mechanical loading is the principal and most extensively investigated pathogenic factor for IVDD. Accumulating studies have revealed that compression could induce NP cell death, which has been believed to play an important role in the pathogenesis of IVDD, via triggering apoptosis and necroptosis and so on [21, 22]. Therefore, preventing NP cells from compression-induced death is an attracting method for IVDD treatment.

TUDCA is a kind of bile salt with ER chaperone-like properties, which can inhibit ER stress that blocks the activation of UPR owing to its ability to modulate ER function [10]. As a clinical drug approved by the FDA, many investigators have demonstrated that TUDCA can protect cells against various stimuli [9, 23]. Seyhun et al. has found that TUDCA could reduce endoplasmic reticulum stress, acinar cell damage, and systemic inflammation in acute pancreatitis [9]. Peng et al. also illustrated that preconditioning with TUDCA protects against contrast-induced HK-2 cell apoptosis [10]. Other studies also proved that TUDCA could hinder the development of osteoarthritis, amyotrophic lateral sclerosis, steatohepatitis, and so on [8, 24, 25]. Therefore, to explore whether this cytoprotective function exists in compression-induced NP cells death, we used TUDCA to treat NP cells. In consistency with previous results, as presented in our article, preconditioning with TUDCA enhanced the survivability of NP cells under excessive compression. The cell population was increased in the presence of TUDCA in a dose-dependent manner. In addition, NP cells treated with high dose of TUDCA showed a healthier morphology than the control group. These results verified that TUDCA had protective effects on NP cells under compression. Moreover, our study suggested that treatment by TUDCA may be a potential way to retard IVDD.

ER stress is a condition that causes disturbances in many homeostatic processes and lead to a state in which protein folding slows [26]. However, the role of ER stress is still controversial because ER stress plays a critical and “double-edged sword-like” role in many diseases. For instance, ER stress plays a detrimental role in cancer but a salutary role in neurodegenerative disease as described in Introduction. Former studies have displayed that ER stress is essential for the pathogenesis of IVDD; thus, targeting the underlying molecular events that regulate the ER stress level of NP cells may offer a new therapeutic strategy for IVDD [17]. In this study, we chose suitable compression to mimic the mechanical pressure of the human L4-L5 disc in a standing position while flexed forward [27], and it was equal to the intensity used in our previous studies. We found that compression could induce ER stress in NP cells, which was reflected by morphological changes of the ER. Exposure to compression of 1 MPa for 18 h resulted in moderate but obvious damage to the ER. The observed damage was more severe and irreversible when the duration was prolonged to 36 h. The Western blot analysis of ER-stress-related biomarkers further confirmed the observation that ER stress was activated after compression. However, the level of p-eIF2*α* did not increase for a compression duration more than 18 h. These results implied that compression-induced ER stress was involved in the pathogenesis of IVDD, and a duration more than 18 h might not further enhance the compression-induced ER stress. Accordingly, we speculated that ER stress was more effective in the middle stage rather than the late stage of IVDD. In an analysis of the apoptosis pathways of human degenerative lumbar discs, the ER pathway was predominantly involved in the mild stage (Pfirrmann grade III) of IVDD [28]. Since TUDCA protects cells from adverse stimulus-induced death via inhibiting ER tress in other diseases, we speculated that TUDCA exerts its cytoprotective effects through the same mechanism. Accordingly, our results showed that preconditioning with TUDCA could attenuate compression-induced ER morphological changes. Furthermore, ER-stress-associated proteins were also downregulated in comparison with the control group. Hence, we could conclude that ER stress takes part in the pathogenesis of compression-induced IVDD and TUDCA could protect NP cells from compression-induced death via inhibiting the ER stress.

Apoptosis is one of the most important mechanisms for human IVD cells loss [29]. Therefore, we want to explore whether TUDCA could inhibit compression-induced NP cells death. Through flow cytometry, fluorescence photomicrographs, and Hoechst 33258 staining, we discovered that compression-induced NP cell apoptosis was dramatically inhibited by TUDCA treatment in comparison with control group. This is in accordance with previous researches demonstrating that TUDCA could protect cells from adverse condition-induced apoptosis. There are three apoptosis signaling pathways, namely, the death receptor pathway, mitochondrial pathway, and ER pathway. All three pathways have been examined with respect to the apoptosis of IVD cells [17, 30]. For the ER pathway of apoptosis, numerous studies have shown that the activation of key molecules, such as CHOP, caspase-12, and cleaved caspase-12, is dependent on the activation of UPR signals. Thus, we assumed that TUDCA decreased the compression-induced apoptosis levels by minimizing UPR signals. In accordance with our hypothesis, the Western blotting results showed that ER pathway apoptosis-related biomarkers including CHOP, caspase-12, and cleaved caspase-12 were all decreased to various level in TUDCA group. These results verified that TUDCA could inhibit the ER pathway of apoptosis induced by compression, which can partly explain why TUDCA showed a protective effect in our research.

The percentage of NP cells with necrotic morphological features increases with age, which testifies that the necrotic cell death of NP cells is precisely controlled by human body [31]. As our recent study revealed, necroptosis is involved in the compression-induced cell death of IVDD and NEC-1 could protect cells from necroptosis in a dose- and time-dependent manner. In addition, it has also been reported that ER stress could induce ligand-independent TNFR1-mediated necroptosis in L929 cells [32]. Considering these facts, we speculated that TUDCA might be able to inhibit compression-induced necroptosis. In our current study, we found that TUDCA could inhibit necroptosis in a dose-dependent manner, which suggested that ER stress might be a major upstream pathway of necroptosis in compression-induced cell death. Recent study has reported that the necroptosis-related proteins, such as mixed lineage kinase domain-like (MLKL) protein and RIPK3, were localized not only at the cell membrane but also on the endoplasmic reticulum of the necroptotic cells [33]. This phenomenon reminded a linkage between ER stress and necroptosis, and inhibition of ER stress could block necroptosis in spinal cord injury. Our study proved that this linkage was also presented in IVDD, but the detailed mechanism of this linkage has to be further researched.

There are several potential limitations related to the present work. First, we only performed monolayer culture of nucleus pulposus cells. However, it is well known that monolayer expansion will change the phenotype of IVD cells, such as decreased gene expression of types II and X collagen and of aggrecan, which might make our results deviate from the real situation [34]. Furthermore, the air pressure used in this study was ten times higher than atmospheric pressure. Although we decreased the concentration of oxygen and carbon dioxide accordingly, which is one-tenth of their concentration in air, this will still more or less influence our results. Therefore, a better* in vitro* model to mimic the degeneration of IVD should be developed in further studies. In addition,* in vivo* animal studies should also be adopted in further researches.

## 5. Conclusion

In conclusion, this is the first study to investigate the protective role of TUDCA in the pathogenesis of IVDD. Our discoveries indicated that TUDCA could protect NP cells from excessive compression-induced death by reducing the apoptosis and necroptosis. In addition, ER stress is involved in the pathogenesis of IVDD induced by excessive compression and plays a detrimental role. TUDCA exerts its protective functions by inhibiting ER stress. Our findings could provide a new method for the therapy of IVDD.
